# Data on the expression of *GSTE1* and *GSTE7* in Drosophila chemosensory organs after isothiocyanate exposure

**DOI:** 10.1016/j.dib.2018.07.062

**Published:** 2018-07-31

**Authors:** Stéphane Fraichard, Daniel Gonzalez, Paul Grassein, Patrice Delarue, Patrick Senet, Adrien Nicolaï, Evelyne Chavanne, Elodie Mucher, Yves Artur, Jean-François Ferveur, Jean-Marie Heydel, Loïc Briand, Fabrice Neiers

**Affiliations:** a Centre des Sciences du Goût et de l׳Alimentation (CSGA), Université de Bourgogne Franche-Comté, INRA, CNRS, France; b Laboratoire Interdisciplinaire Carnot de Bourgogne, UMR 6303 CNRS-Univ. Bourgogne Franche-Comté, 9 Av. A. Savary, BP 47 870, F-21078 Dijon Cedex, France

## Abstract

The data presented in this article are related to the research article entitled “Characterization of a Drosophila glutathione transferase involved in isothiocyanate detoxification.” (Gonzalez et al., 2018) [1]. This article includes the expression level of *Drosophila melanogaster GSTE1* and *GSTE7* in chemosensory male tissues and the expression level of the mRNAs coding for the same enzymes after a PEITC exposure in food.

**Specifications Table**

**Table:** 

Subject area	Biology
More specific subject area	Toxicology
Type of data	Figure
How data was acquired	RT-qPCR
Data format	Analyzed
Experimental factors	Chemosensory organs were prepared from flies exposed or no to PEITC.
Experimental features	*Drosophila melanogaster* antennae, palps, labellum and forelegs
Data source location	Dijon, France
Data accessibility	Data are supplied with this article

**Value of the data**

• The data presented in this article show that *GSTE1* and *GSTE7 mRNA* are expressed in male chemosensory tissues.
• *GSTE1* and *GSTE7* mRNA expression is significantly higher in antennae and palps compared to heads. *GSTE1* mRNA expression is higher in labellum and forelegs compared to Drosophila heads.
• A three day-long exposure to food containing PEITC led to a significant increase of *GSTE7* mRNA expression in taste organs but did not significantly change *GSTE1* mRNA expression in chemosensory tissues.

## 1 Data

**Fig. 1:**
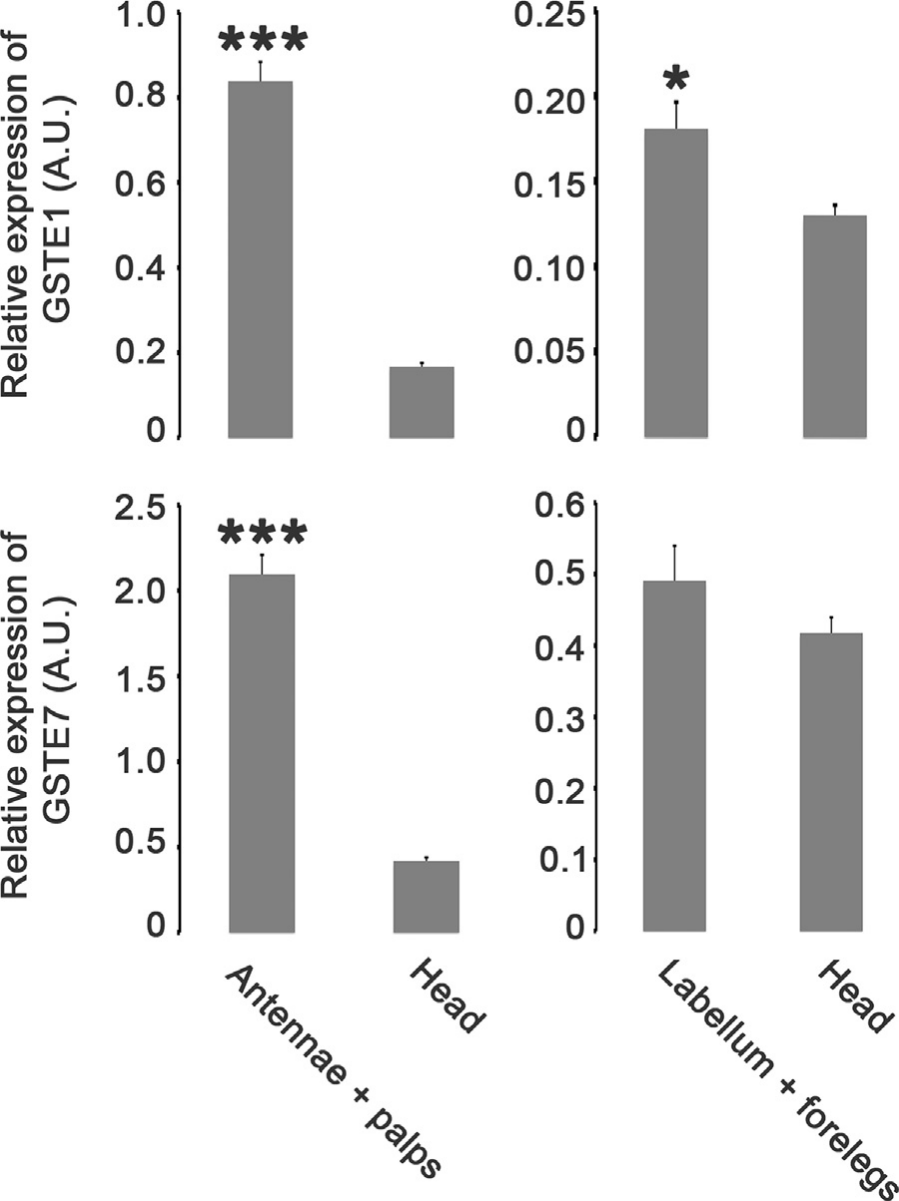
Expression level of *D. melanogaster GSTE1* and *GSTE7* in male chemosensory tissues. Real time PCR analysis was performed using RNA extracted from olfactory appendages (antennae and palps), taste appendages (labellum and forelegs) and heads deprived of chemosensory appendages. The numbers shown on the y-axis represent arbitrary units indicating relative level of the RNAs.

**Fig. 2:**
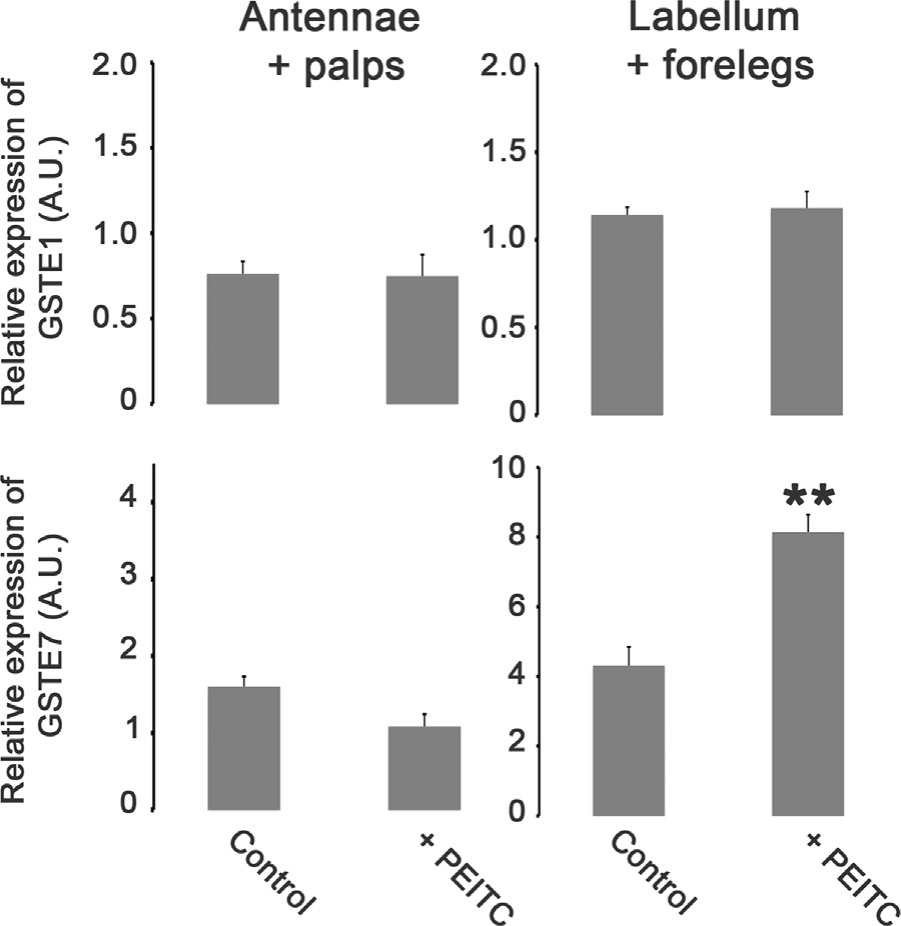
Effects of PEITC on expression of *GSTE1* and *GSTE7* RNAs in chemosensory organs of adult male flies. Relative expression of *GSTE1* and *GSTE7* RNAs in olfactory appendages (antennae + palps), in taste appendages (labellum + forelegs) from Drosophila males exposed to PEITC (0.25 mM) or control. The numbers shown on the y axis are arbitrary units indicating relative level of the RNAs.

The data shown here describe the *GSTE1* and *GSTE7* mRNA expression in Drosophila male chemosensory organs and are related to the research article entitled “Characterization of a Drosophila glutathione transferase involved in isothiocyanate detoxification.” (Gonzalez et al., 2018) [1]. The relative amount of mRNAs coding for *GSTE1* and *GSTE7* showed a higher expression level in olfactory organs (antennae and palps) compared to fly heads (Fig. 1). After a three day-long exposure to food containing phenethyl isothiocyanate (PEITC), only the *GSTE7* mRNA expression level was changed (Fig. 2). This exposure led to an increased expression in the labellum and forelegs.

## 2 Experimental design, materials and methods

### 2.1 Drosophila strains, rearing conditions and ITC treatments

For this study, we used Canton-S (Cs) wild-type male flies. Flies were reared on standard yeast/cornmeal/agar medium in a humidified, temperature-controlled incubator at 25 °C under a 12 h light: 12 h dark cycle.

PEITC (CAS no. 2257-09-2) was dissolved in ethanol (final concentration of PEITC was 0.25 mM) and added to the media at 50 °C (Merck, Kenilworth, New Jersey, USA). A similar volume of ethanol was added for both the experimental and drug-free control tests. Flies were transferred to experimental treatments at a density of 10 per vial. 30 flies were used in each treatment and they were exposed to experimental treatments during 3 successive days.

### 2.2 RNA extraction and RT-qPCR

Total RNA was extracted using Isol RNA Lysis reagent (5Prime) and was treated with RNAse-free DNAse (Euromedex, Souffelweyersheim, France) to avoid genomic DNA contamination. Total RNA was reverse-transcribed using the iScript cDNA Synthesis Kit (BioRad, Hercules, USA). The qPCR reactions were carried out on a MyIQ (BioRad, Hercules, USA) using the IQ SYBR Green SuperMix (BioRad, Hercules, USA). Each reaction was performed in triplicate. All results were normalized relatively to the tubulin and rp-49 mRNA levels and the relative amount of mRNAs were calculated using the ∆∆Ct method.

All error bars represent SEMs. REST Software was used to compare qPCR sets of data. Asterisks indicate the level of statistical significance (* *p* <0.05, ** *p* <0.01, *** *p* <0.001) [2].
